# Hyperglycemic Stress Induces Expression, Degradation, and Nuclear Association of Rho GDP Dissociation Inhibitor 2 (RhoGDIβ) in Pancreatic β-Cells

**DOI:** 10.3390/cells13030272

**Published:** 2024-02-01

**Authors:** Noah Gleason, Anjaneyulu Kowluru

**Affiliations:** 1 Research Service, John D. Dingell VA Medical Center, Detroit, MI 48201, USA; gp8205@wayne.edu; 2 Department of Pharmaceutical Sciences, Eugene Applebaum College of Pharmacy and Health Sciences, Wayne State University, Detroit, MI 48201, USA

**Keywords:** RhoGDIβ, small G proteins, insulin secretion, islet β-cell

## Abstract

Small G proteins (e.g., Rac1) play critical regulatory roles in islet β-cell function in health (physiological insulin secretion) and in metabolic stress (cell dysfunction and demise). Multiple regulatory factors for these G proteins, such as GDP dissociation inhibitors (GDIs), have been implicated in the functional regulation of these G proteins. The current set of investigations is aimed at understanding impact of chronic hyperglycemic stress on the expression and subcellular distribution of three known isoforms of RhoGDIs (RhoGDIα, RhoGDIβ, and RhoGDIγ) in insulin-secreting β-cells. The data accrued in these studies revealed that the expression of RhoGDIβ, but not RhoGDIα or RhoGDIγ, is increased in INS-1 832/13 cells, rat islets, and human islets. Hyperglycemic stress also promoted the cleavage of RhoGDIβ, leading to its translocation to the nuclear compartment. We also report that RhoGDIα, but not RhoGDIγ, is associated with the nuclear compartment. However, unlike RhoGDIβ, hyperglycemic conditions exerted no effects on RhoGDIα’s association with nuclear fraction. Based on these observations, and our earlier findings of the translocation of Rac1 to the nuclear compartment under the duress of metabolic stress, we conclude that the RhoGDIβ-Rac1 signaling module promotes signals from the cytosolic to the nucleus, culminating in accelerated β-cell dysfunction under metabolic stress.

## 1. Introduction

Glucose-stimulated (physiological) insulin secretion (GSIS) from the pancreatic β-cell involves significant interplay between metabolic and cationic events, leading to the generation of second messenger molecules (inositol triphosphates, cyclic, adenine, and guanine nucleotides), which are needed for the activation of signaling events that lead to the translocation of insulin-laden secretory granules to the plasma membrane for the fusion and secretion of insulin [1,2,3,4]. The activation of a variety of GTP-binding proteins (G proteins), including those belonging to the Rho (Rac1, Cdc42), Rab (Rab 3A, Rab27), and Arf (Arf6) subfamilies, has been implicated as one of the key signaling steps involved in GSIS [5,6,7]. The (de)activation of these G proteins is under the control of a variety of factors, including the GTP/GDP exchange factors (GEFs), GDP-dissociation inhibitors (GDIs), and GTPase-activating proteins (GAPs) [8,9,10]. From a mechanistic standpoint, the GEFs facilitate the activation of G proteins by promoting the release of GDP to enable GTP binding. The GAPs catalyze the hydrolysis of GTP-bound G proteins to GDP, resulting in the inactivation of the candidate G proteins [8,10,11,12]. The functional roles of the GDIs include the sequestration of G proteins in their GDP-bound conformation, thereby preventing their functional activation by GEFs. In this context, the G protein activation–deactivation (also referred to as the GTPase hydrolytic cycle) mediated by these proteins/factors is highly complex given the fact that many GEFs (~82) and GAPs (~70) are expressed in mammalian cells [11,13]. Interestingly, despite such a large abundance of GEFs and GAPs, only three GDIs, namely, RhoGDIα, RhoGDIβ, and RhoGDIγ, are expressed in mammalian cells. RhoGDIα (also known as RhoGDI1) is expressed ubiquitously. RhoGDIβ (also referred to as RhoGDI2, LyGDI, D4-GDI, and ARHGDIB) has been shown to be expressed in hematopoietic cells. RhoGDIγ (also known as RhoGDI3) has been shown to be expressed predominantly in the brain, pancreas, lungs, kidney, and testis [9,11,13,14,15]. Recent investigations by Ahmad Mokhtar and coworkers suggested that RhoGDIα and RhoGDIβ bind and regulate functions of the Rho and Rac subfamilies of G proteins, whereas RhoGDIγ exhibits a wider specificity of regulation of typical G proteins such as Rho, Rac, and Cdc42, as well as some atypical G proteins such as Wrch2/RhoV, Rnd2, Miro2, and RhoH [13].

In the context of our current understanding of the putative regulatory roles of RhoGDIs in islet β-cell function in health and metabolic stress (the focus of the current studies), earlier studies from our laboratory demonstrated the key roles for RhoGDIα in insulin secretion from pancreatic β-cells. We demonstrated that the overexpression of wild type GDI markedly attenuated glucose-induced, but not KCl- or mastoparan-induced, insulin secretion from pancreatic β-cells. The siRNA-mediated depletion of RhoGDIα augmented GSIS in these cells, suggesting an inhibitory role for RhoGDIα in the glucose metabolic signaling cascade, including the activation of Rac1, which is requisite for GSIS to occur [6]. More recent investigations from our laboratory revealed participatory roles of RhoGDIβ in islet β-cell function. We demonstrated that the siRNA-mediated knockdown of RhoGDIβ in INS-1 832/13 cells significantly attenuated glucose-induced Rac1 activation without affecting its translocation and membrane association. Further, the suppression of RhoGDIβ expression exerted minimal effects on GSIS at the height of the inhibition of Rac1 activation, suggesting paradoxical and divergent effects of RhoGDIβ on Rac1 activation and insulin secretion in the glucose-stimulated β-cell [16]. More importantly, data from our investigations suggested that, in contrast to the traditional roles of RhoGDIs playing inhibitory roles of G protein activation, it appears that RhoGDIβ contributes to the glucose-induced activation of Rac1 in the pancreatic β-cell [16]. Our findings provide further strength to earlier observations in other cell types (e.g., Jurkat cells, myofibroblasts) demonstrating the stimulation of Rac1 by RhoGDIβ [17,18,19]. Altogether, there appear to be clear differences between RhoGDIα and RhoGDIβ in terms of their ability to mediate GSIS. To the best of our knowledge, putative roles of RhoGDIγ in insulin secretion have not been addressed to date.

In the context of functional roles of RhoGDIs in the regulation of cellular events leading to cellular dysfunction, a growing body of evidence implicates RhoGDIβ in the pathology of various forms of cancers. Interestingly, the expression of RhoGDIβ has been reported to either increase or decrease in different forms of cancer [11]. RhoGDIβ has also been shown to exert roles in several cellular functions, including actin cytoskeletal organization, immune response, vascular remodeling, and cellular apoptosis [11,14]. From a mechanistic standpoint, earlier studies have reported the caspase-3 (or a caspase-3-like protease)-mediated cleavage of RhoGDIβ at Asp19, leading to the translocation of the truncated form of RhoGDIβ to the nucleus for the propagation of signals necessary for cell apoptosis [20,21,22,23]. Studies conducted by Essman and coworkers in BJAB Burkitt-like lymphoma cells revealed that RhoGDIβ, but not RhoGDIα, is cleaved by caspase-3 during drug-induced apoptosis [24]. Together, these observations implicate a critical role for caspase-3 in the regulatory control of RhoGDIβ function and subcellular distribution in a variety of pathological states. Despite this evidence in other cell types, significant knowledge gaps exist with regard to its regulatory roles in islet β-cell function under the conditions of metabolic stress. Therefore, the current set of investigations is undertaken to determine the potential alterations, if any, in the expression of RhoGDIβ in pancreatic β-cells following exposure to chronic hyperglycemic conditions. We also ask whether RhoGDIβ is degraded under these conditions and whether it would impact the subcellular (cytosolic vs. nuclear) distribution consequential to exposure to hyperglycemic stress.

## 2. Materials and Methods

### 2.1. Chemicals and Reagents

Antibodies specific for RhoGDIα (sc-373724), RhoGDIβ (sc-271108), RhoGDIγ (sc-393690), cleaved RhoGDIβ (sc-52936), and Lamin B (sc-56144) were obtained from Santa Cruz Biotechnology (Dallas, TX, USA). GAPDH (5174S) antisera and HRP-conjugated secondary antibodies were obtained from Cell Signaling (Danvers, MA, USA). β-actin (A1974) antibody was acquired from Sigma Aldrich (St. Louis, MO, USA). The NE-Per nuclear and cytoplasmic isolation kit was purchased from Thermo Fisher Scientific (Waltham, MA, USA).

### 2.2. INS-1 832/13 Cells, MIN6 Cells, Rodent Islets, and Human Islets

INS-1 832/13 β-cells were obtained from Sigma Aldrich (St. Louis, MO, USA). MIN6 β-cells were obtained from AddexBio (San Diego, CA, USA). Pancreatic islets were isolated from Sprague Dawley rats by the collagenase digestion method we described earlier [16]. All protocols were approved by the Institutional Animal Care and Use Committees at Wayne State University and the John D. Dingell VA Medical Center. Human islets were acquired from Prodo Laboratories (Aliso Viejo, CA, USA). Approval of studies involving human islets was acquired from the Biosafety Committee of John D. Dingell VA Medical Center.

### 2.3. Cell Culture and Experimental Conditions

INS-1 832/13 cells, rat islets, and human islets were cultured in RPMI-1640 media containing 11.1 mM glucose, 10% heat-inactivated fetal bovine serum (FBS), 100 IU/mL penicillin and 100 IU/mL streptomycin, 1 mM sodium pyruvate, 50 µM β-mercaptoethanol (not included for medium used with rat and human islets), and 10 mM HEPES (pH 7.4–7.6). MIN6 cells were cultured in Dulbecco’s Modified Eagle Medium (DMEM; 25 mmol/L glucose) supplemented with 15% heat-inactivated FBS, 2 mmol/L L-Glutamine, 100 IU/mL penicillin and 100 IU/mL streptomycin, and 50 µM β-mercaptoethanol [25,26]. Cells were starved overnight in a low-glucose/low-serum growth medium (2.5 mM glucose and 2.5% FBS) overnight before glucose treatment conditions (2.5 mM or 20 mM) were initiated for the specified time periods as indicated in the text.

### 2.4. Western Blotting

Following incubation under the specified experimental conditions, cell lysates were separated by SDS-PAGE and transferred onto nitrocellulose membranes, and the membranes were blocked for 1 h at room temperature with 3% BSA in PBS-T (0.1%). This was followed by the overnight incubation of the membranes in the primary antibody for proteins of interest at 4 °C (1:1000 dilution for RhoGDIs; 1:5000 dilution for β-actin, Lamin B, and GAPDH). Following the removal of the primary antibodies, the membranes were washed and probed with appropriate secondary HRP-conjugated antibodies (1:2000) in 1.5% PBS-T for 1 h at room temperature. Secondary antibodies were removed, and target proteins were detected by chemiluminescence and band intensities were quantified by densitometry using Image Studio Lite software (V5.2; LI-COR Biosciences; Lincoln, NE, USA).

### 2.5. Isolation of Cytosolic and Nuclear Fractions

INS-1 832/13 cells were incubated under basal (LG; 2.5 mM) or hyperglycemic stress (HG; 20 mM) conditions for 45 min or 24 h prior to the isolation of cytosolic and nuclear fractions, which was performed using the NE-PER Extraction kit as per the manufacturer’s protocol described earlier [27]. The purity of these fractions was assessed by the determination of specific marker proteins, namely, GAPDH and Lamin B for the cytosolic and nuclear fractions, respectively.

### 2.6. Confocal Imaging

INS-1 832/13 cells were incubated (for 24 h) with either basal (LG; 2.5 mM) or high (HG; 20 mM) glucose on chamber slides. Samples were prepared for confocal imaging according to previously published protocols from our laboratory [28]. Images were obtained by a 63×oil objective utilizing a Zeiss Axio Examiner Z1 upright microscope. Image analysis was completed using Volocity 7.0 software [27]. All confocal microscopy studies were conducted at the Microscopy, Imaging, and Cytometry Resources Core at Wayne State University School of Medicine.

### 2.7. Statistical Analysis

An analysis of the difference between the control and treatment groups was performed using GraphPad Prism version 9.5 (GraphPad Software, San Diego, CA, USA). Data were presented as mean ± SEM from three or more independent experiments. Comparisons between two groups were analyzed with a two-tailed Student’s *t*-test. A *p*-value below 0.05 was considered significant.

## 3. Results

Earlier investigations from our laboratory demonstrated that the incubation of INS-1 832/13 cells to hyperglycemic conditions (HG; 20 mM; 24 h) culminates in mitochondrial dysfunction (e.g., caspase-3 activation) and nuclear abnormalities (e.g., nuclear Lamin B degradation) leading to impaired GSIS, loss of metabolic cell viability, and β-cell demise [29]. Therefore, we utilized this experimental model in the following investigations to determine the potential impact of HG conditions on the expression and subcellular distribution of RhoGDIs in pancreatic β-cells.

**Figure 1:**
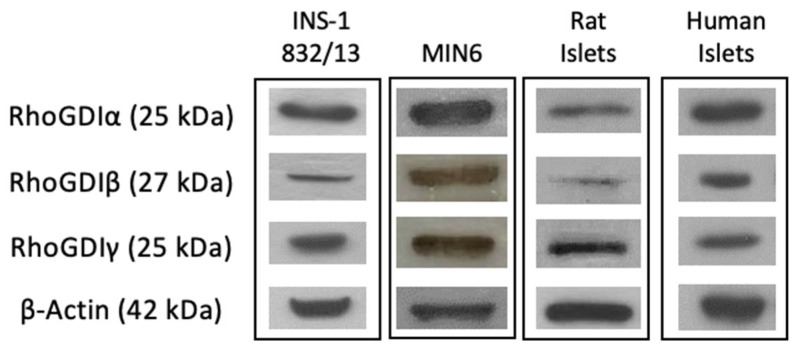
RhoGDI expression in INS-1 832/13 cells, MIN6 cells, rat islets, and human islets. Western blot data depicting the expression of RhoGDIα, RhoGDIβ, and RhoGDIγ in INS1 832/13 cells, MIN6 cells, rat islets, and human islets.

**Figure 2:**
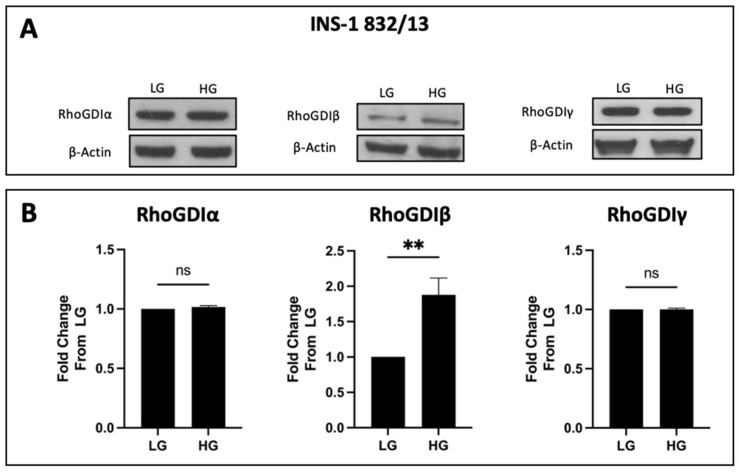
Expression levels of RhoGDIα, RhoGDIβ, and RhoGDIγ in INS-1 832/13 cells exposed to either basal or chronic hyperglycemic conditions. (**A**): Representative Western blots from multiple studies showing the effect of high-glucose conditions on the expression of RhoGDIα (n = 3), RhoGDIβ (n = 7), and RhoGDIγ (n = 3). Cells were cultured in the presence of either basal (LG; 2.5 mM) or high-glucose (HG; 20 mM) treatment for 24 h and the degree of expression of RhoGDIα, RhoGDIβ, and RhoGDIγ was determined by Western blotting. β-actin was used as a loading control. (**B**): Densitometric quantification of RhoGDIα, RhoGDIβ, and RhoGDIγ using Image Studio Lite v3.1 (Li-COR; Lincoln, Nebraska). Data are presented as mean ± SEM with values shown as fold change from LG; significance is considered for *p*-values < 0.05 (**: *p*-value < 0.01) ns = not significant.

**Figure 3:**
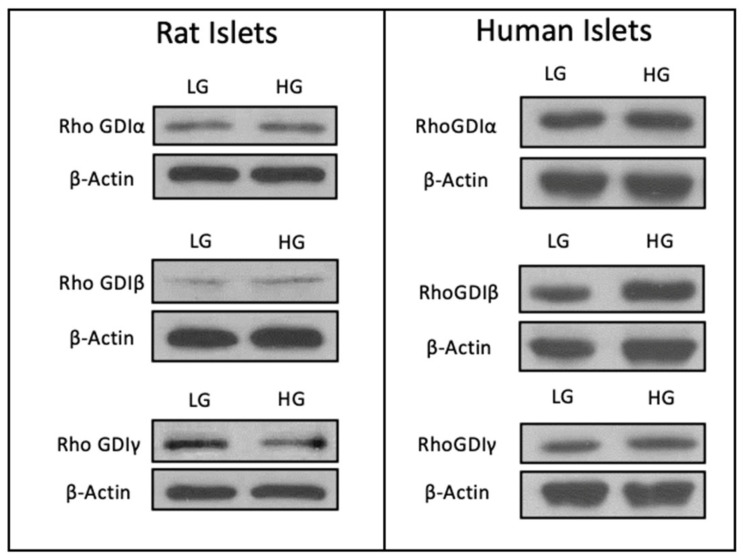
Expression levels of RhoGDIα, RhoGDIβ, and RhoGDIγ in rat and human islets exposed to either basal or chronic hyperglycemic conditions. Representative Western blots from rodents and human islets showing the expression of RhoGDIα, RhoGDIβ, and RhoGDIγ when exposed to 24 h basal (LG; 2.5 mM) or high glucose (HG; 20 mM). β-actin was used as a loading control. Rat islet data shown are from a single study (n = 1), while human islet data are from 2 separate studies.

To address this question, we determined the relative abundance of all three forms of RhoGDIs (RhoGDIα, RhoGDIβ, and RhoGDIγ) in a variety of insulin-secreting cells, including INS-1 832/13 cells, MIN6 cells, rat islets, and human islets. The data shown in Figure 1 demonstrate that all three forms of GDIs are expressed in these four types of insulin-secreting cells that were examined. As stated above, while a growing body of evidence in multiple cell types, including the islet β-cell, implicates novel regulatory roles for small G proteins belonging to the Rho subfamily (e.g., Rac1) in the onset of cell dysfunction under pathological conditions (e.g., metabolic stress), the potential regulatory effects of metabolic stress on the expression and subcellular distribution of the three RhoGDIs in pancreatic β-cells remain unknown. To address this question, we determined the expression levels of RhoGDIα, RhoGDIβ, and RhoGDIγ in INS-1 832/13 cells, rat islets, and human islets incubated (for 24 h) under basal (2.5 mM) and hyperglycemic (20 mM) conditions. The data accrued from these investigations indicated minimal effects of hyperglycemic conditions on the expression of RhoGDIα and RhoGDIγ in INS-1 832/13 cells (Figure 2) and rat islets and human islets (Figure 3). However, we noticed a significant increase in the expression of RhoGDIβ in all of the cells exposed to hyperglycemic stress (Figure 2 and Figure 3).

**Figure 4:**
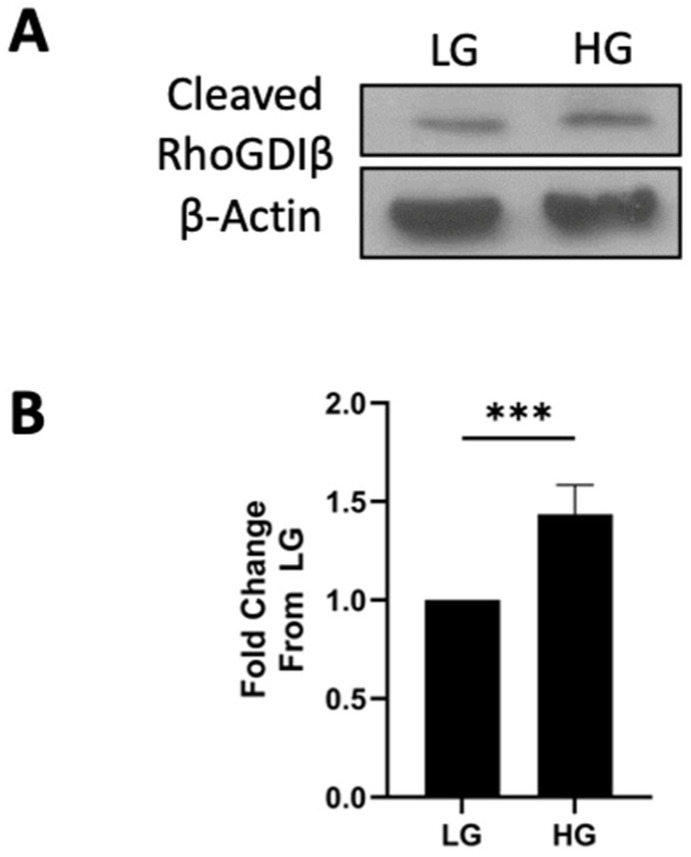
Chronic hyperglycemic conditions increase the cleavage of RhoGDIβ in INS-1 832/13 cells. (**A**): A representative Western blot displaying cleaved RhoGDIβ expression in INS-1 832/13 lysates exposed to basal (LG; 2.5 mM) or high-glucose (HG; 20 mM) conditions for 24 h is shown here. β-actin was used as a loading control. (**B**): Densitometric quantification of expression levels of cleaved RhoGDIβ in INS-1 832/13 cells following exposure to either basal (LG; 2.5 mM) or high glucose (HG; 20 mM) for 24 h. Data are presented as mean ± SEM. Results are represented as fold change from LG (n = 5; ***: *p* < 0.001).

The findings from several laboratories demonstrated the increased expression of RhoGDIβ under various pathological conditions, including certain forms of cancers [11]. Furthermore, it has been shown that RhoGDIβ undergoes caspase-3-mediated degradation at Asp19, resulting in the increased generation of the cleaved form of RhoGDIβ (Δ19-RhoGDIβ), which has been implicated in various cellular processes, including alterations in cell polarity [30]. In addition, as stated above, the proteolytic cleavage of RhoGDIβ by caspase-3 has been implicated in cellular apoptosis [21]. Since it has been shown that chronic metabolic stress induces mitochondrial defects culminating in cytochrome C release and the subsequent activation of caspase-3 in pancreatic β-cells, we asked whether RhoGDIβ is degraded in pancreatic β-cells under the duress of glycemic stress. To address this, using specific antisera, we determined the relative abundance of both forms of RhoGDIβ (i.e., full length and the cleaved forms) in INS-1 832/13 cells incubated under basal and chronic hyperglycemic conditions. The data shown in Figure 4 indicate that, in addition to the increased expression of RhoGDIβ (Figure 2), we saw a significant increase in the levels of cleaved RhoGDIβ in INS-1 832/13 cells incubated under hyperglycemic conditions (Figure 4A). The data from multiple experiments are plotted in Figure 4B.

**Figure 5:**
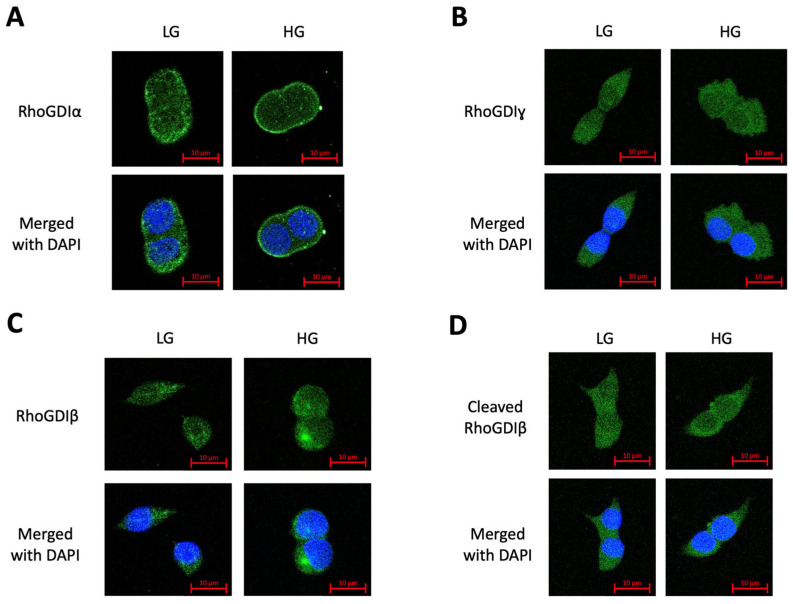
Confocal imaging analysis of the expression of RhoGDIα, RhoGDIγ, and RhoGDIβ (full-length and cleaved forms) in INS-1 832/13 cells. Representative confocal images displaying the expression and subcellular distribution of RhoGDIα (**A**), RhoGDIγ (**B**), full-length RhoGDIβ (**C**), and cleaved RhoGDIβ (**D**) using FITC conjugated secondary antibody (green) in INS-1 832/13 cells exposed to either basal (LG; 2.5 mM) or high-glucose (HG; 20 mM) conditions for 24 h, as described in the text. DAPI is shown as a marker for the nuclear association (blue). Images shown are representative of 2 independent confocal studies.

As stated above, existing studies, specifically in the cancer field, have reported the association between cleaved/truncated RhoGDIβ and the nuclear fraction. Therefore, in the next set of experiments, we examined, via confocal imaging, the subcellular distribution of RhoGDIα, RhoGDIγ, and RhoGDIβ (full-length and cleaved) in INS-1 832/13 cells exposed to basal and hyperglycemic conditions. The data in Figure 5 highlight observations from these investigations. First, under basal conditions, RhoGDIα appeared to be diffusely distributed throughout the cell (Figure 5A). Interestingly, the exposure of these cells to chronic hyperglycemic stress promoted the movement of RhoGDIα toward the plasma membrane. No evidence of nuclear association (i.e., colocalization with DAPI) of RhoGDIα was seen in these cells following exposure to high-glucose conditions (Figure 5A; lower panel).

The data shown in Figure 5B appear to suggest that RhoGDIγ is diffusely distributed throughout the cell under both basal and hyperglycemic exposure conditions with no evidence of colocalization with DAPI (i.e., nuclear association). Interestingly, however, we noticed that hyperglycemic conditions facilitate the association of RhoGDIβ (full-length) in the perinuclear region (Figure 5C) and RhoGDIβ in the cleaved form (i.e., increased granularity in the DAPI stained region; Figure 5D). Together, these findings appear to provide directional, but not definitive, evidence for a potential association between RhoGDIβ (full-length and cleaved forms), but not RhoGDIα or RhoGDIγ, and the nuclear compartment.

**Figure 6:**
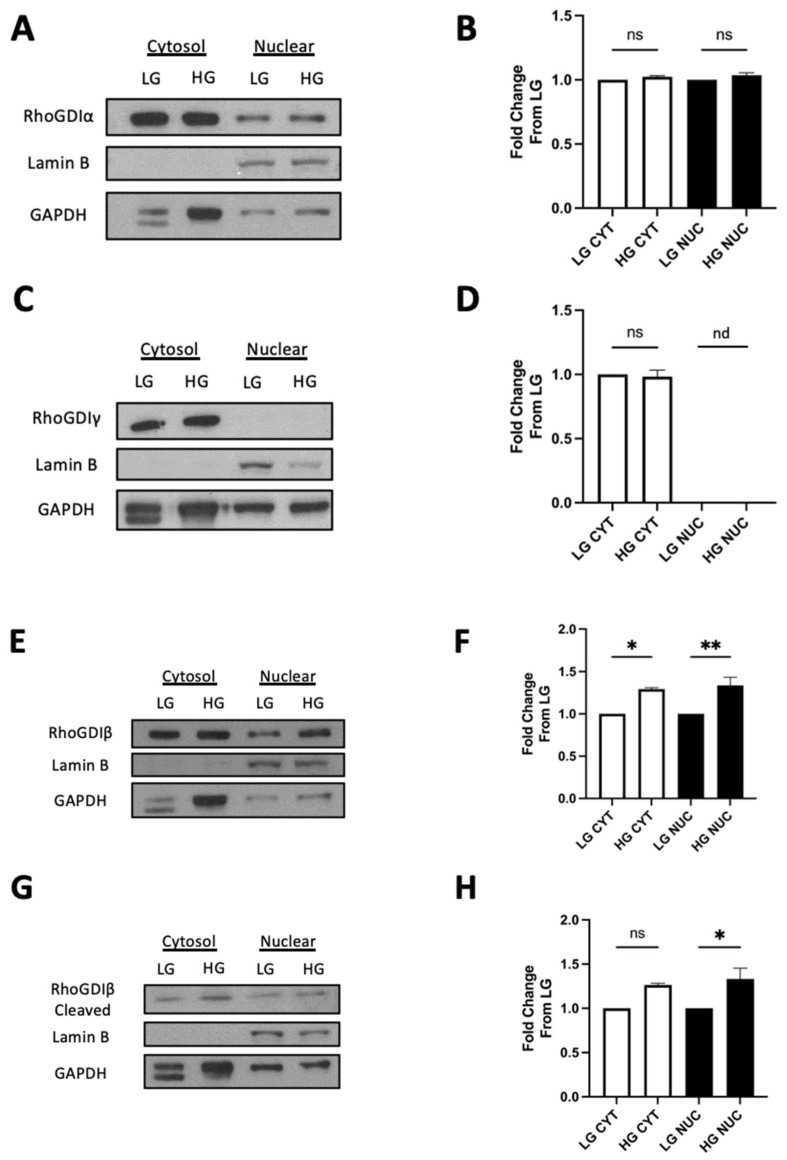
Non-nuclear and nuclear association of RhoGDIα, RhoGDIγ, RhoGDIβ (full-length and cleaved) in INS-1 832/13 cells exposed to basal or hyperglycemic conditions. A representative Western blot depicting the association of RhoGDIα (**A**) and RhoGDIγ (**C**). Densitometric analysis of cytosol (CYT) and nuclear (NUC) expression for RhoGDIα (**B**) and RhoGDIγ (**D**) in INS-1 832/13 cells exposed to either basal (LG; 2.5 mM) or high-glucose (HG; 20 mM) conditions for 24 h from 3 independent studies (n = 3; nd: not detected). Representative Western blots showing the expression of full-length RhoGDIβ (**E**) and cleaved RhoGDIβ (**G**) in INS-1 832/13 cells exposed to either basal (LG; 2.5 mM) or high-glucose (HG; 20 mM) conditions for 24 h are shown here. Densitometric analysis of pooled data are presented for full-length RhoGDIβ ((**F**), n = 3) and cleaved RhoGDIβ ((**H**), n = 3). *: *p*-value < 0.05, **: *p*-value < 0.01. GAPDH and Lamin B were used as loading and purity controls for the cytosolic and nuclear fractions, respectively. Data are expressed as fold change from LG; significance considered when *p* < 0.05. ns = not significant; nd = not detected.

In the next set of investigations, we attempted to further validate this formulation via the immunoblotting approach. To address this, we isolated cytosolic and nuclear fractions from INS-1 832/13 cells incubated under basal and hyperglycemic conditions using a commercially available kit and determined the relative abundance of proteins in question in these fractions via Western blotting (see Methods for additional details). The data shown in Figure 6A indicate that a significant portion of RhoGDIα is localized in the cytosolic fraction with no significant effects of hyperglycemic conditions on the relative distribution of this protein in the cytosolic and nuclear fraction (Figure 6B). Interestingly, RhoGDIγ appears to reside exclusively in the cytosolic fraction under both basal and hyperglycemic conditions (Figure 6C,D). The data depicted in Figure 6E,F suggest a significant increase in the nuclear association of RhoGDIβ (full-length protein) in cells exposed to glycemic stress. Furthermore, we noticed a significant increase in the association between the cleaved form of RhoGDIβ and the nuclear fraction in cells under the duress of glycemic stress (Figure 6G,H).

**Figure 7:**
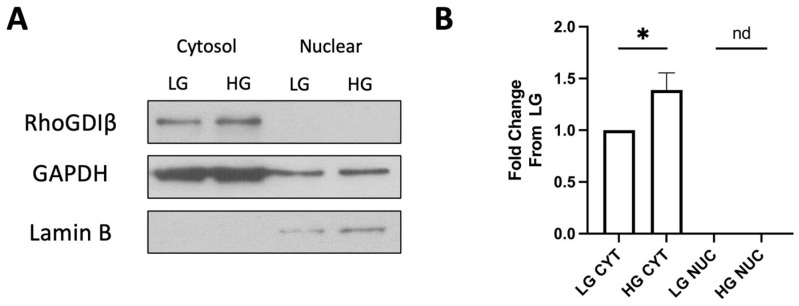
Examination of non-nuclear and nuclear association of full length RhoGDIβ in INS-1 832/13 cells exposed acutely to either basal or stimulatory glucose. Representative Western blots depicting relative abundance of full-length RhoGDIβ (**A**) in INS-1 832/13 cells exposed to either basal (2.5 mM) or stimulatory glucose (20 mM) for 45 min are shown here. Densitometric analyses of data from multiple experiments highlighting abundance in the cytosolic (CYT) and nuclear (NUC) fractions for full-length RhoGDIβ (**B**) are shown. GAPDH and Lamin B were used as markers for the cytosolic and nuclear fractions, respectively. Data are expressed as fold change from LG; *: *p* < 0.05 (n = 3). nd = not detected.

Lastly, to further validate that the nuclear association of RhoGDIβ is not due to a physiological response to stimulatory glucose under short-term conditions (acute effects), we determined the relative abundance of RhoGDIβ in non-nuclear and nuclear fractions isolated from INS-1 832/13 cells exposed to basal (2.5 mM) and stimulatory glucose (20 mM; 45 min). The data shown in Figure 7A,B demonstrate no significant translocation of RhoGDIβ to the nuclear fraction under acute regulatory conditions by the stimulatory concentration of glucose. In fact, we observed a modest, but significant, increase in the expression of RhoGDIβ in the cytosolic fraction derived from HG-treated cells. These data suggest that the translocation of RhoGDIβ to the nuclear compartment in β-cells exposed to chronic hyperglycemic conditions might represent a key signaling step involved in the loss of β-cell function under metabolic stress conditions (see below).

## 4. Discussion

The overall objective of our current studies was to determine the potential impact of hyperglycemic conditions on the expression and subcellular localization of the three known GDIs for Rho G proteins in human islets, rodent islets, and clonal β-cells. This has not been addressed before. The data accrued from these investigations revealed that RhoGDIα, RhoGDIβ, and RhoGDIγ are expressed in these cells, and that the expression of RhoGDIβ, but not RhoGDIα or RhoGDIγ, is increased under the duress of hyperglycemic stress. Furthermore, these studies revealed that RhoGDIβ undergoes cleavage under metabolic stress, resulting in its translocation to the nucleus. The potential significance of these findings in the context of the metabolic-stress-induced dysfunction of the islet β-cell is discussed below.

Published evidence regarding multiple cell types suggests that the physiological functions of Rac1 are regulated by RhoGDIβ [6,31]. While this appears to be true in the pancreatic β-cell, more recent evidence appears to indicate paradoxical roles for RhoGDIβ in islet β-cell function following exposure to glucose under acute as well as chronic exposure conditions. First, studies by Thamilselvan and coworkers demonstrated that the siRNA-mediated knockdown of RhoGDIβ markedly reduced glucose-induced Rac1 activation, without affecting its membrane association. In addition, the siRNA-mediated depletion of RhoGDIβ elicited minimal roles in GSIS. Taken together, these observations are suggestive of functional roles of RhoGDIβ as a positive modulator of the glucose-induced activation of Rac1 in pancreatic β-cells since the depletion of RhoGDIβ inhibited the glucose-induced activation of Rac1 [16]. The available evidence in other cell types (as highlighted above) affirms roles for RhoGDIβ as a stimulator of small G proteins, including Rac1. Clearly, such functions of RhoGDIβ are paradoxical, since RhoGDIs are known to impede G protein activation. For example, studies by Zhang and coworkers demonstrated the constitutive activation of Rac1 and its downstream signaling steps, including p38MAPK activation in MDA-MB-231 cells following the depletion of RhoGDIβ [32]. Potential regulatory mechanisms that underlie the positive and negative modulatory roles of RhoGDIβ in G protein activation and cell function currently remain unclear. They might include the regulation of RhoGDIβ function via phosphorylation at different sites. For example, it has been shown that the phosphorylation of RhoGDIβ at Tyr153 by Src kinase leads to the inhibition of metastasis, in contrast to phosphorylation by protein kinase-α at Ser31, which promotes metastasis [33,34]. Therefore, additional investigations are necessary for a better understanding of the roles of RhoGDIβ in the regulation of G protein in/activation and cellular function.

In addition to its increased expression, we also demonstrated the nuclear association of full-length as well as cleaved forms of RhoGDIβ in pancreatic β-cells under the duress of metabolic stress. In addition, our Western blot data indicated the presence of RhoGDIα in the nuclear fraction even though metabolic stress conditions did not increase its association with the nuclear fraction. To our knowledge, this is the first evidence in support of the nuclear association between G protein regulatory factors (e.g., RhoGDIs) and the nuclear fraction under chronic metabolic stress conditions in pancreatic β-cells. Evidence in many cell types, including various forms of cancer and UV radiation, are suggestive of the caspase-3-mediated cleavage of RhoGDIβ, leading to its translocation to the nuclear fraction. The potential implications of the increased nuclear association of RhoGDIβ in various pathological conditions remain unclear even though several postulations have been put forth. For example, Choi and coworkers proposed that the caspase-3-mediated cleavage of RhoGDIβ expedites the progression of apoptosis in HL60 and K562 leukemia cells [22]. Studies by Krieser and Eastman suggested that the caspase-3-induced cleavage of RhoGDIβ in ML-1 cells leads to the activation of Jun N-terminal kinase, which is an upstream regulator of apoptosis [21]. The data accrued from the studies of Zhou et al. suggested that Trp53-dependent ionizing radiation-induced apoptosis in thymus cells involves the translocation of caspase-3-cleaved RhoGDIβ, but not the full-length form, to the nuclear fraction. Interestingly, these investigators presented data to suggest that the mechanisms underlying cleaved-RhoGDIβ-mediated apoptosis do not involve Rho G proteins, since the latter were not present in the nuclear fraction [20]. Based on these findings, the researchers concluded that the cleavage and association of RhoGDIβ with the nuclear fraction enables the transmission of signals from the cytoplasm to the nucleus to promote cell apoptosis [20]. While these postulations are important for cell dysfunction and demise in the stated cells, there appears to be some key differences between the above studies and the data from our current investigations. First, we noted an association between both the full-length and cleaved RhoGDIβ and the nuclear fraction under metabolic stress. Interestingly, the abundance of only full-length, but not cleaved, RhoGDIβ is increased in the nuclear fraction derived from high-glucose-treated cells. Second, we may not be able to rule out the regulation of G proteins (e.g., Rac1) by the full-length and cleaved RhoGDIβ in the nuclear fraction, since we have reported tthe translocation of Rac1 to the nuclear fraction in pancreatic β-cells following exposure to metabolic stress [6]. Additional studies, including the potential metabolic-stress-induced translocation of RhoGDIβ to the nuclear fraction derived from rat and human islets, are needed to precisely define the roles of RhoGDIβ (full-length and cleaved forms) in the modulation of the signals needed for metabolic-stress-induced islet β-cell dysfunction.

Lastly, RhoGDIβ might partake in additional signaling events, leading to islet β-cell dysfunction under conditions of metabolic stress. For example, recent investigations by Gamage and coworkers suggested that RhoGDIβ remains complexed with Rac1 under basal glucose conditions, and that it dissociates from Rac1 under chronic hyperglycemic conditions via its complexation with Caspase Recruitment Domain-Containing Protein 9 (CARD9), a scaffolding protein implicated in innate immunity [29]. Mechanistically, these studies revealed that the increased complexation of RhoGDIβ with CARD9 results in the activation of Rac1 and its downstream signaling events, including p38MAPK, leading to β-cell dysregulation. Together, these findings suggested that RhoGDIβ elicits multiple regulatory effects on G protein function. In addition to its association with other scaffolding proteins, it is also likely that RhoGDIβ might facilitate the activation of specific G proteins (e.g., Rac1) via interactions with specific GEFs [31,35,36,37,38]. Indeed, evidence from the studies of Groysman and associates on interaction between RhoGDIβ and Vav proteins (known GEFs for Rac1) provides additional insights into the potential crosstalk between GEFs and GDI in the regulation of cell function [39]. Additional studies are needed to precisely understand the roles of RhoGDIβ in islet function in health (i.e., GSIS) and metabolic stress (i.e., cell dysregulation and demise).

Our current studies also revealed the nuclear association of RhoGDIα in pancreatic β-cells. However, HG conditions had minimal effects on its nuclear association. The potential roles of RhoGDIα in the nuclear compartment remain to be investigated further in pancreatic β-cells. Along these lines, using the proteomics approach, Sandrock and coworkers identified RhoGDIα as an interacting partner for Rac1 in the nuclear fraction derived from HeLa cells [40]. Additional studies are needed to further assess the roles of nuclear RhoGDIα in islet β-cell function. Lastly, the data from our studies demonstrated no association between RhoGDIγ and the nuclear fraction isolated from LG- or HG-treated cells. Interestingly, studies by Brunet and coworkers reported an association between RhoGDIγ and the Golgi apparatus, and implicated this GDI in the delivery of specific G proteins (RhoG) to relevant subcellular compartments [41]. The potential roles of RhoGDIγ in islet function remain unknown. It is likely that it might be involved in RhoG-Rac1 signaling steps, leading to β-cell dysfunction under metabolic stress. This remains to be verified.

In conclusion, we provide the first evidence for the specific effects of metabolic stress on the expression, cleavage, and nuclear association of RhoGDIβ in pancreatic β-cells. Based on the existing data from other cell types and emerging data from pancreatic β-cells, it appears to contribute more toward G protein regulation and the propagation of signals from the cytosolic compartment to the nuclear compartment, leading to cellular dysfunction. Interestingly, it seems to mediate additional functional roles such as binding to scaffolding proteins such as CARD9 under the duress of metabolic stress, thereby facilitating the sustained activation of Rac1, culminating in the activation of stress kinases (p38MAPK) and NADPH oxidases (Nox2) to induce cell dysfunction and demise under a variety of experimental conditions, including metabolic stress [29,31,42].

## Data Availability

Data will be made available on request.
